# The molecular epidemiology of HIV-1 in Sweden 1996 to 2022, and the influence of migration from Ukraine

**DOI:** 10.2807/1560-7917.ES.2023.28.48.2300224

**Published:** 2023-11-30

**Authors:** Anna Weibull Wärnberg, Johanna Brännström, Olof Elvstam, Magnus Gisslén, Fredrik Månsson, Anders Sönnerborg, Maarten AA van de Klundert

**Affiliations:** 1 Department of Infectious Diseases, Karolinska University Hospital, Stockholm, Sweden; 2 Department of Infectious Diseases, Södersjukhuset, Stockholm, Sweden; 3 Division of Infectious Diseases, Department of Medicine Huddinge, Karolinska Institutet, Stockholm, Sweden; 4 Department of Infectious Diseases, Växjö Central Hospital, Växjö, Sweden; 5 Department of Translational Medicine, Clinical Infection Medicine, Lund University, Malmö; 6 Department of Infectious Diseases, Institute of Biomedicine, Sahlgrenska Academy, University of Gothenburg, Gothenburg, Sweden; 7 Region Västra Götaland, Department of Infectious Diseases, Sahlgrenska University Hospital, Gothenburg, Sweden; 8 Division of Clinical Microbiology, Department of Laboratory Medicine, Karolinska Institutet, Stockholm, Sweden

**Keywords:** HIV subtypes, HIV-1A6, Sweden, Ukraine, molecular epidemiology

## Abstract

**Background:**

The global distribution of HIV-1 subtypes is evolving, which is reflected in the Swedish HIV cohort. The subtype HIV-1A6, which may be prone to developing resistance to cabotegravir, is the most common subtype in Ukraine.

**Aim:**

We aimed to examine trends in HIV-1 subtype distribution in Sweden, with a special focus on HIV-1A6, and to describe the virology, demography and treatment of Ukrainian people living with HIV (PLWH) who migrated to Sweden in 2022.

**Methods:**

Data about PLWH in Sweden are included in a national database (InfCareHIV). We used the online tool COMET to establish HIV-1 subtypes and the Stanford database to define drug resistance mutations. We investigated the relation between virological characteristics and demographic data.

**Results:**

The early epidemic was predominated by HIV-1 subtype B infections in people born in Sweden. After 1990, the majority of new PLWH in Sweden were PLWH migrating to Sweden, resulting in an increasingly diverse epidemic. In 2022, HIV-1A6 had become the sixth most common subtype in Sweden and 98 of the 431 new PLWH that were registered in Sweden came from Ukraine. We detected HIV RNA in plasma of 32 Ukrainian patients (34%), of whom 17 were previously undiagnosed, 10 had interrupted therapy and five were previously diagnosed but not treated. We found HIV-1A6 in 23 of 24 sequenced patients.

**Conclusion:**

The molecular HIV epidemiology in Sweden continues to diversify and PLWH unaware of their HIV status and predominance of HIV-1A6 should be considered when arranging care directed at PLWH from Ukraine.

**Key public health message**

**What did you want to address in this study and why?**

We wanted to monitor changes in HIV-1 diversity in Sweden. It is important to follow the spread of variants, such as the subtype HIV-1A6. This subtype is common in eastern part of the World Health Organization European Region and should be monitored because it may easily become resistant to innovative long-acting injectable HIV medication. We focused on people who had migrated from Ukraine so the care for them can be adjusted to their needs.

**What have we learnt from this study?**

The distribution of HIV-1 subtypes in Sweden has changed a lot since the beginning of the AIDS epidemic and has become very diverse, mostly due to migration of already infected people to Sweden. Currently, HIV-1A6 is the sixth most common subtype. People from Ukraine are not always aware that they are infected, and the majority of Ukrainian people living with HIV are infected with HIV-1A6.

**What are the implications of your findings for public health?**

It is important to perform HIV sequence analysis to observe the spread of new subtypes. People originating from Ukraine should be encouraged to be tested for HIV. Standard care using Swedish protocols proved to be effective for the treatment of Ukrainian people living with HIV. The high percentage of individuals infected with subtype HIV-1A6 should be taken in account when considering therapy with long-acting injectable HIV medication.

## Introduction

Knowledge of the molecular epidemiology of human immunodeficiency virus type 1 (HIV-1) infection and the presence of pre-treatment drug resistance are important for HIV-1 surveillance and for effective use of antiretroviral therapy (ART). The HIV-1 epidemic in Europe and the Americas was initially caused by subtype B (HIV-1B), whereas subtype A (HIV-1A) predominated in several regions in Africa and in countries that belong to the eastern part of the WHO European Region in the early years of the AIDS epidemic. However, the global subtype distribution has changed [1]. In addition, circulating recombinant forms (CRFs) and unique recombinant forms (URFs) have increased and currently account for approximately one fifth of all HIV-1 infections globally.

Western Europe and North America attained a reduction in new HIV cases by 16% between 2010 and 2021 [2]. In contrast, the situation in parts of eastern Europe and Russia is poorly controlled and the number of new infections increased by 48% in the same period [2]. Russia has the highest HIV-1 burden in Europe with ca 1 million people infected, followed by Ukraine with 260,000 PLWH. Approximately 68% of PLWH in Ukraine are reported to be aware of their HIV-positive status and among them, 59% are receiving ART [3]. The HIV-1 epidemic in eastern Europe and Russia, countries that at the time formed the Soviet Union, began in the mid-1990s after a single introduction of an HIV-1A virus in people who inject drugs (PWID) in Ukraine. The circulating virus became a phylogenetically distinct outgroup is now classified as HIV-1A6 [4]. The HIV-1A6 virus still accounts for more than 85% of all infections in the FSU [4]. The predominant route of infection in Ukraine and Russia has shifted from injection drug use to heterosexual transmission, although it should be noted that men who have sex with men (MSM) as a transmission route may be under-reported due to cultural stigma. While HIV-1B infections used to predominate and remain relatively common in Russian and Ukrainian MSM, HIV-1A6 currently accounts for the majority of infections also in this population [4].

Sweden has a low prevalence of HIV (78/100,000 persons in 2022) [5]. In earlier studies we showed that the epidemiology evolved from 1983 to 2012 from being dominated by HIV-1B infections in Swedish-born MSM and PWID, to a predominance of PLWH born outside of Sweden that were infected with non-B subtypes by heterosexual transmission [6-8]. The Swedish National HIV Registry (InfCareHIV), which includes more than 99% of PLWH in Sweden, reported in 2023 that two thirds of the Swedish PLWH in active care are born abroad [9].

It has been reported that HIV-1A6 is more prone to developing resistance to the long-acting injectable integrase strand transfer inhibitor (INSTI) cabotegravir (CAB) [10], which has been approved in an increasing number of countries since 2021. The combination of CAB and rilpivirine (RPV) has shown efficient viral suppression in multiple large non-inferiority studies [11], but HIV-1A6 stands out as a risk factor for virological failure, particularly in people with a body mass index > 30 kg/m^2^ and pre-existing RPV resistance [10]. Recently, the World Health Organization has recommended long-acting injectable CAB for use in pre-exposure prophylaxis.

A novel promising therapy are broadly neutralising antibodies (BNAbs). A recent phase IB study showed effective viral suppression when administered in combination with lenacapavir (a long-acting capsid inhibitor) every 6 months [12]. Importantly, the effectiveness of such antibodies is highly specific for the envelope of one or more HIV-1 subtypes.

Here, we describe the temporal distribution of the HIV-1 subtypes in Sweden from 1996 to 2022, with special focus on HIV-1A6. We describe the demographics of PLWH who migrated to Sweden from Ukraine after February 2022. Especially, we focus on HIV subtypes and drug resistance mutations (DRMs), infection awareness and antiretroviral treatment of PLWH before and after leaving Ukraine.

## Methods

### Study population and data processing

InfCareHIV is a prospective national cohort including more than 99% of PLWH in Sweden [9]. The InfCareHIV database was established in 2003 and by 2008, all HIV care centres in Sweden were affiliated. Data on patients diagnosed before 2003 has been added retrospectively [13]. Since 2008, patients have been included at their first visit to a Swedish HIV clinic. Demographic, laboratory and therapeutic data are updated in real-time when patients visit their HIV clinic. The most probable country of infection and route of transmission are self-reported at enrolment. We extracted cohort data from the InfCareHIV database on 16 February 2023. Data on migration were derived from the Swedish Migration Agency database (extracted on 30 January 2023). 

Ukrainian migrants without any information on treatment within the 30 days preceding registration were classified as ‘not treated’, unless they had HIV-1 RNA < 50 copies/mL which we defined as ‘probably treated’. Patients with no record of treatment and HIV-1 RNA ≥ 50 copies/mL and 2022 as ‘year of diagnosis’ were assumed to be newly diagnosed in Sweden. 

Patient data and data to assign geographical regions to the country of birth and country of acquisition were imported to a custom database in R using customised scripts and the RStudio terminal [14]. Details regarding the applied definition of countries and regions are available in the Supplement. Statistical analyses were performed and visualised using RStudio, Microsoft Excel and GraphPad Prism.

Since 1996, the protease and reverse transcriptase have been sequenced by Sanger sequencing in most patients with sufficient HIV RNA load [7,8]. More recently, the integrase gene has also been routinely sequenced. Subtypes were determined using the programme HIV-1 COMET [15]. For classification of DRMs, we used the Stanford HIV drug resistance database (HIVdb; version 9.1) [16]. In the present study, we defined ‘virologically suppressed’ as < 50 HIV-1 RNA copies/mL to account for previous assays with varying detection limits. For phylogenetic analysis, only sequences of at least 900 bp with a known sampling date were used. Phylogenetic analysis was performed and visualised using the Nextstrain workflow [17]. The settings for Nextstrain were similar to the settings applied in our earlier study [4].

## Results

### Study population

**Figure 1:**
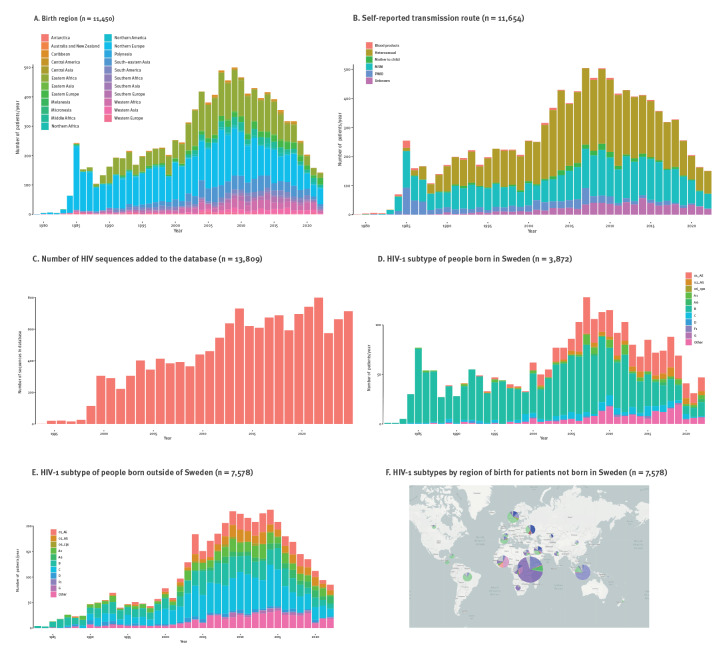
Characteristics of people living with HIV, by year of diagnosis, Sweden, 1996–2022

MSM: men who have sex with men; PWID: people who inject drugs.

In Sweden, HIV RNA quantification and pol gene sequence analysis became standard practice in 1996, while HIV RNA concentration and sequence data before 1996 may not be representative due to a more pronounced selection bias. Therefore, we excluded PLWH who had died, emigrated from Sweden or were lost to follow up before 1 January 1996, and analysed only data from PLWH (n = 11,654) who were enrolled between 1 January 1996 and 31 December 2022. On 31 December 2022, 8,549 PLWH were registered as patients receiving care at the time at an HIV clinic in Sweden. For 11,450 PLWH, the birth country was registered and of these, 7,578 (66.2%) were born abroad (Table 1 and Figure 1A). During the study period, the predominant route of infection shifted from MSM- and PWID-related infections in people born in Sweden, to heterosexual transmission in people who had migrated to Sweden (Figure 1A,B). Of 3,872 people born in Sweden, 2,285 (59.0%) were infected in Sweden and 1,146 (29.6%) were infected abroad; for the remaining 441 (11.4%), country of infection was unknown (Table 1).

**Table 1:** Demographic data of people living with HIV-1 enrolled in Swedish healthcare, 1996–2022 (n = 11,654)

	Swedish-born		Born outside Sweden		Overall^a^	
	n	%	n	%	n	%
Total	3,872	33.2	7,578	65.0	11,654	100
Sex^b^						
Male	3,233	83.5	4,089	54.0	7,455	64.0
Female	639	16.5	3,479	45.9	4,184	35.9
Missing	0	0	10	0.1	15	0.1
Age in years at diagnosis						
Mean (SD)	39.7 (13.0)		33.0 (11.3)		35.3 (12.3)	
Median (min–max)	38 (0–90)		32 (0–91)		34 (0–91)	
Missing	33	0.9	183	2.4	280	2.4
Infected in or after arrival in Sweden						
Yes	2,285	59.0	781	10.3	3,072	26.4
No	1,146	29.6	6,115	80.7	7,275	62.4
Missing	441	11.4	682	9.0	1,307	11.2
First HIV-1 RNA load						
Below LOQ^c^	166	4.3	1,530	20.2	1,727	14.8
Above LOQ^c^	3,666	94.7	5,939	78.4	9,740	83.6
Missing	40	1.0	109	1.4	187	1.6
First HIV-1 RNA load (log10)^d^						
Mean (SD)	4.64 (1.09)		4.48 (1.09)		4.54 (1.09)	
Median (min–max^e^)	4.72 (1.70–6.00)		4.61 (1.70–6.00)		4.65 (1.70–6.00)	
First CD4^+^ T-cell count (cells/μL)						
Mean (SD)	459 (327)		387 (310)		412 (318)	
Median (min–max)	420 (0–5,730)		340 (0–4,660)		369 (0–5,730)	
Missing	25	0.6	97	1.3	152	1.3
Transmission route						
Blood products	52	1.3	112	1.5	167	1.4
Heterosexual	1,298	33.5	4,276	56.4	5,621	48.2
PWID	534	13.8	265	3.5	819	7.0
Mother to child	33	0.9	255	3.4	288	2.5
MSM	1,844	47.6	1,765	23.3	3,652	31.3
Unknown or missing	111	2.9	905	11.9	1,107	9.5

LOQ: limit of quantification; MSM: men having sex with men; PWID: people who inject drugs; SD: standard deviation.

^a^ Overall also includes 204 people with missing data on birth country.

^b^ Biological sex at birth, as a binary variable. Information about transgender status was introduced in the InfCareHIV database in 2021 and is not presented in the table.

^c^ LOQ = 50 copies/mL.

^d^ In patients with HIV RNA > LOQ only.

^e^ Upper limit of quantification: 10^6^ copies/mL.

The circle size in Panel F corresponds to the number of migrants from the region.

### Molecular epidemiology of HIV-1 infection in Sweden between 1996 and 2022

In 1996, HIV-1 RNA testing and sequence analysis became routine in Swedish HIV care, resulting in a large increase in the number of available sequences (Figure 1C). On 31 December 2022, the InfCareHIV database contained 13,809 HIV-1 sequences from 7,274 individuals (n = 11,564 protease and/or reverse transcriptase, n = 2,029 integrase and n = 216 envelope). All sequences were obtained from viraemic individuals at diagnosis or from pre-treated individuals who were viraemic due to therapy intermission or failure. The 10 most common HIV-1 subtypes were B (n = 2,562), C (n = 1,467), 01_AE (n = 988), A1 (n = 556), 02_AG (n = 399), A6 (n = 209), D (n = 154), G (n = 94), 06_cpx (n = 48), and F1 (n = 26) (Figure 1D-F).

The early Swedish HIV epidemic was characterised by almost exclusively HIV-1B infections (Figure 1D,E). During the peak of new diagnoses in 2008 and 2009, HIV-1B only accounted for 214 of 991 (22%) infections and by 2022, the number of newly included patients infected with HIV-1B had further dropped to 25 of 160 (16%) (Figure 1D,E). This change was primarily attributable to the increase from 2002 onwards in PLWH who were born outside Sweden (Figure 1A,D,E). We observed a clear relation between HIV-1 subtype and country of birth (Figure 1F). In line with global regional differences in subtype prevalence, HIV-1A1 sequences and HIV-1C were mostly found in people from eastern Africa, HIV-1G and HIV-102_AG were more common in western Africa, HIV-1B was predominantly found in people from Europe and the Americas and HIV-101_AE was most common in South East Asia, while HIV-1A6 was almost exclusively found in migrants from eastern Europe and the FSU.

### Phylogenetic analysis

**Figure 2:**
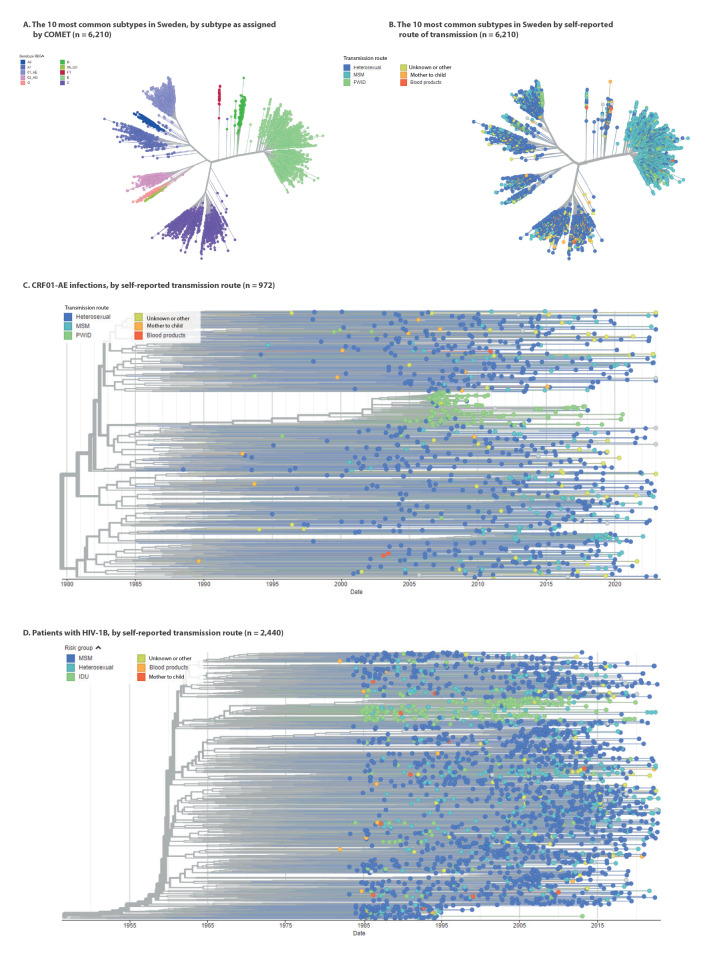
Phylogenetic analysis of unique patient sequences of the major HIV-1 subtypes circulating in Sweden, 1996–2022 (n = 6,210)

MSM: men who have sex with men; PWID: people who inject drugs.

We generated a phylogenetic tree of the 10 most common subtypes circulating in Sweden. The subtypes formed clear clusters, indicating correct subtyping (Figure 2A). Assigning colours to the sequences based on transmission route indicated that HIV-1B was associated with transmission within MSM (Figure 2B). The phylogenetic analysis also revealed two PWID-related sub-clusters in other subtypes. The first was related to the well-described outbreak of HIV-1 CRF01_AE in Sweden and Finland in 2006 and 2007 (Figure 2C) [18]. After 2007, 61 new cases related to this cluster were identified, the last three as recent as 2020 (Figure 2C; data not shown). The second PWID-related sub-cluster was a phylogenetically distinct cluster in the HIV-1B cluster (Figure 2D).

### HIV-1A6 in Sweden, years 1996 to 2022

**Figure 3:**
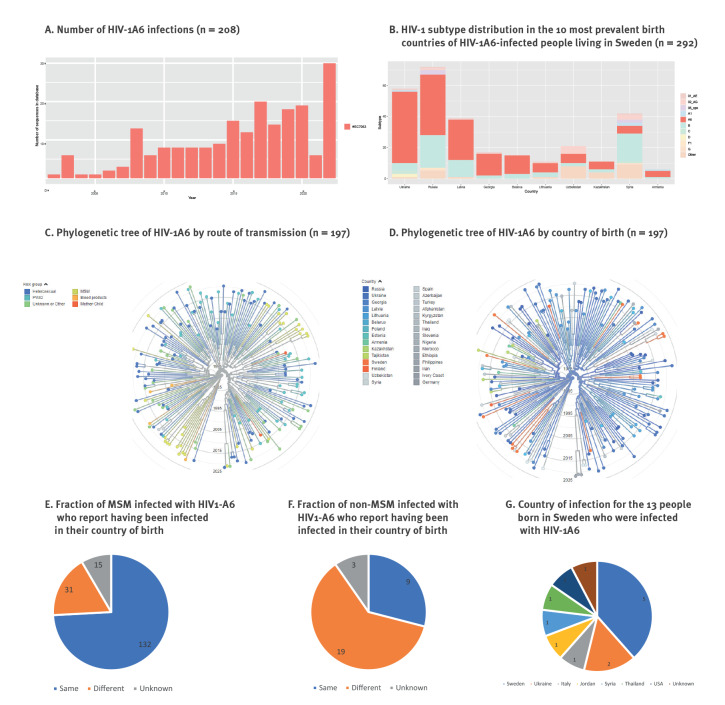
HIV-1A6 prevalence, Sweden, 2000–2022

MSM: men who have sex with men; PWID: people who inject drugs

The first case of HIV-1A6 infection in Sweden that we identified was registered in 2000 (Figure 3A). Since then, the number of new HIV-1A6 infections has gradually increased, mostly due to migration from countries where it is the predominant subtype (Figure 3B). We provide a detailed overview in Supplementary Table S1 of the demographic data and subtypes of the patients from the countries from which most HIV1-A6 patients came to Sweden. Among those, we identified in total 208 unique HIV-1A6 sequences in PLWH in Sweden. As no subtype information is available for patients with undetectable plasma HIV-1 RNA, the 208 sequences of confirmed HIV1-A6 cases are likely to represent an underestimation of the total number of HIV1-A6 cases in Sweden. Based on the fraction of PLWH with HIV-1A6 among those with available sequences per country, multiplied by the total number of PLWH from these countries in the InfCareHIV database, we estimated that a total of 391 PLWH infected with HIV-1A6 are or have been treated in Swedish healthcare between 2000 and 2022. 

Subtype HIV-1A6 formed a distinct cluster within the HIV-1A cluster (Figure 2A) [4]. A more detailed analysis of all HIV-1A6 sequences in patients followed in Sweden revealed that there were also two clusters related to MSM (Figure 3C). These clusters were not related to birth country (Figure 3D), year of infection (Figure 3C,D), or country of infection. A visualisation of the phylogenetic relation between HIV-1A6 isolates and country of infection has been made available in the Supplement.

Most HIV-1A6 infections (165/208) were identified among PLWH born in the FSU and infected in their home country (Figure 3B and 3E). A notable exception were MSM, who were more frequently infected in countries other than their birth country (Figure 3F). Of the 13 Swedish-born PLWH with HIV-1A6, five were infected in Sweden, two in Ukraine and six in other (non-FSU) countries (Figure 3G).

### Drug resistance mutations in HIV-1A6 strains

All protease and reverse transcriptase sequences of the 208 HIV-1A6-infected patients were analysed for DRMs. In total 14 (6.7%) of HIV-1A6 had one or more nucleoside analogue reverse transcriptase inhibitor (NRTI) DRM. The A62V polymorphism, that was previously described to be widespread in HIV-1A6 [4] and that does not substantially affect NRTI susceptibility on its own but is often found in combination with DRMs, was identified in 44 (21%) of our HIV-1A6 cases. Non-nucleoside RTI (NNRTI) DRM were present in 26 (13%) isolates of which E138A with eight (3.9%) cases (one in combination with V106VI) was the most prevalent and may reduce RPV susceptibility [19]. We included a record of less common individual DRMs in Supplementary Table S2. Three PLWH infected with HIV-1A6 had multiple DRMs against both NRTI and NNRTI. These were all born in FSU countries and were viraemic when entering HIV care in Sweden. Four isolates with protease inhibitor (PI) DRMs were identified, three with M46I and one with M46I, I54L and L90M. The latter had also the NRTI M184V DRM and one of the former three M46I mutants carried a V108VI (NNRTI) DRM. Another three patients had strains with two NRTI and/or NNRTI DRM. We made a record of all identified multidrug-resistant isolates available in Supplementary Table S3.

For 91 HIV-1A6 patients, the corresponding integrase sequences were available and of these, 90 were pure A6. No integrase DRMs were identified, but in line with earlier observations, the L74I polymorphism was found in 87 of 90 sequences A6 integrase sequences. One virus that had an A6 protease and reverse transcriptase gene had a HIV-1A1 integrase sequence, thus this virus was most probably a unique recombinant form (URF) between HIV-1A6 and HIV-1A1. 

### People living with HIV who migrated from Ukraine to Sweden in 2022

In 2022, an estimated 7.9 million Ukrainian citizens migrated to countries in the European Union, 51,263 of them to Sweden. Health examination including testing for HIV, tuberculosis and hepatitis B and C was offered on arrival, but unfortunately the percentage of people from Ukraine who underwent screening is not registered. Seventeen Ukrainian migrants who were not previously aware of their HIV status tested positive for HIV-1. These newly diagnosed had a median age of 42 years (interquartile range (IQR): 35–47), a median CD4^+^ T-cell count of 210 cells/μL (IQR: 80–498), and 11 of them were women. We provide a detailed overview of their demographics in Supplementary Table S4. Another 81 individuals self-reported HIV-positive status. Thus, 98 Ukrainian PLWH who migrated to Sweden in 2022 were identified, making up 23% (98/431) of the new PLWH registered in Sweden in 2022 (Table 2).

**Table 2:** Demographic and virological characteristics of people living with HIV, by country of origin, who were registered in a Swedish health clinic for the first time in 2022, Sweden (n = 431)

	Ukraine (n = 98)		Sweden (n = 61)		Thailand (n = 21)		Ethiopia (n = 11)		Eritrea (n = 10)		Kongo (n = 10)		Russia (n = 10)		Sudan (n = 9)		Uganda (n = 9)		Cameroon (n = 8)		Other (n = 184)		Overall (n = 431)	
	n	%	n	%	n	%	n	%	n	%	n	n	n	%	n	%	n	%	n	%	n	%	n	%
Sex ^a^																								
Man	36	36.7	49	80.3	5	23.8	6	54.5	4	40.0	4	40.0	9	90.0	6	66.7	1	11.1	7	87.5	124	67.4	251	58.2
Woman	60	61.2	12	19.7	12	57.1	5	45.5	6	60.0	6	60.0	1	10.0	3	33.3	8	88.9	1	12.5	55	29.9	169	39.2
Missing	2	2.0	0	0.0	4	19.0	0	0.0	0	0.0	0	0.0	0	0.0	0	0.0	0	0.0	0	0.0	5	2.7	11	2.6
Age in years at diagnosis																								
Mean (SD)	35.4 (9.72)		45.8 (14.4)		38.2 (14.6)		39.8 (12.2)		38.7 (14.5)		33.6 (15.6)		28.3 (9.98)		30.6 (9.79)		32 (16.1)		36.5 (9.43)		33.5 (12.7)		36.1 (13.1)	
Median (min–max)	35 (10.0–60.0)		47.5 (20.0–69.0)		34 (17.0–64.0)		40 (8.00–52.0)		41 (5.00–56.0)		37 (0–51.0)		26.5 (17.0–42.0)		29 (14.0–47.0)		33 (4.00–58.0)		32.5 (27.0–52.0)		32 (0–77.0)		34 (0–77.0)	
Missing	7	7.1	5	8.2	2	9.5	1	9.1	0	0	1	10.0	2	20.0	0	0	2	22.2	0	0	21	11.4	41	9.5
Infected in Sweden or after arrival in Sweden																								
Yes	0	0	34	55.7	2	9.5	0	0	1	10.0	0	0	2	20.0	1	11.1	0	0	0	0	18	9.8	58	13.5
No	91	92.9	20	32.8	18	85.7	11	100	7	70.0	9	90.0	6	60.0	8	88.9	9	100	7	87.5	146	79.3	332	77.0
Missing	7	7.1	7	11.5	1	4.8	0	0	2	20.0	1	10.0	2	20.0	0	0	0	0	1	12.5	20	10.9	41	9.5
Transmission route																								
Blood products	4	4.1	0	0	0	0	0	0	0	0	0	0	0	0	0	0	0	0	0	0	4	2.2	8	1.9
Heterosexual	50	51.0	32	52.5	11	52.4	8	72.7	7	70.0	5	50.0	2	20.0	2	22.2	5	55.6	3	37.5	60	32.6	185	42.9
Mother to child	1	1.0	0	0	0	0	1	9.1	1	10.0	2	20.0	0	0	1	11.1	1	11.1	0	0	4	2.2	11	2.6
MSM	4	4.1	23	37.7	5	23.8	0	0	0	0	1	10.0	3	30.0	5	55.6	0	0	5	62.5	76	41.3	122	28.3
PWID	7	7.1	0	0	0	0	0	0	0	0	0	0	2	20.0	0	0	0	0	0	0	0	0	9	2.1
Unknown or other	0	0	0	0	0	0	0	0	0	0	0	0	0	0	0	0	0	0	0	0	0	0	0	0
Missing	32	32.7	6	9.8	5	23.8	2	18.2	2	20.0	2	20.0	3	30.0	1	11.1	3	33.3	0	0	40	21.7	96	22.3
First CD4^+^ T-cell count																								
Mean (SD)	530 (320)		371 (271)		289 (278)		457 (251)		342 (261)		377 (358)		601 (295)		472 (224)		493 (193)		530 (148)		480 (272)		464 (287)	
Median (min–max)	505 (0–1,460)		340 (2.00–1,000)		218 (0–1,110)		430 (170–990)		230 (100–850)		240 (120-1,120)		800 (39.0–920)		450 (67.0–771)		510 (164–704)		534 (320–780)		470 (0–1,310)		450 (0–1,460)	
Missing	6	6.1	3	4.9	0	0	0	0	1	10.0	2	20.0	1	10.0	0	0	0	0	0	0	15	8.2	28	6.5
First RNA load < 50 copies/mL																								
Yes	63	64.3	6	9.8	9	42.9	4	36.4	3	30.0	4	40.0	3	30.0	4	44.4	7	77.8	6	75.0	93	50.5	202	46.9
No	32	32.7	54	88.5	12	57.1	7	63.60	6	60.0	5	50.0	6	60.0	5	55.6	2	22.2	2	25.0	84	45.7	215	49.9
Missing	3	3.1	1	1.6	0	0	0	0	1	10.0	1	10.0	1	10.0	0	0%	0	0	0	0	7	3.8	14	3.2
First RNA load (log 10)^b^																								
Mean (SD)	4.29 (1.49)		5.09 (1.13)		4.62 (1.41)		4.33 (1.23)		4.9 (1.83)		4.39 (0.532)		3.53 (1.34)		3.67 (1.51)		4.79 (1.56)		3.94 (2.63)		4.6 (1.41)		4.62 (1.38)	
Median (min–max)	4.71 (1.74–6.75)		5.38 (1.88–6.98)		4.99 (1.90–6.19)		4.79 (1.76–5.49)		4.42 (2.80–8.00)		4.43 (3.77-4.94)		3.95 (1.72–5.00)		4.09 (1.93–5.60)		4.79 (3.69–5.90)		3.94 (2.08–5.80)		4.82 (1.75-7.00)		4.83 (1.72–8.00)	
Missing	66	67.3	7	11.5	9	42.9	4	36.4	4	40.0	5	50.0	4	40.0	4	44.4	7	77.8	6	75.0	101	54.9	217	50.3
HIV-1 subtype																								
A6	23	23.5	3	4.9	0	0	0	0	0	0	0	0	3	30.0	0	0	0	0	0	0	3	1.6	32	7.4
B	1	1.0	14	23.0	1	4.8	0	0	0	0	0	0	1	10.0	0	0	0	0	0	0	16	8.7	33	7.7
G	0	0	1	1.6	0	0	0	0	0	0	1	10.0	0	0	0	0	0	0	1	12.5	0	0	3	0.7
C	0	0	2	3.3	1	4.8	6	54.5	4	40.0	2	20.0	0	0	0	0	0	0	0	0	13	7.1	28	6.5
D	0	0	0	0	0	0	0	0	1	10.0	0	0	0	0	1	11.1	0	0	0	0	1	0.5	3	0.7
01_AE	0	0	15	24.6	7	33.3	0	0	1	10.0	0	0	0	0	0	0	0	0	0	0	5	2.7	28	6.5
A1	0	0	4	6.6	0	0	0	0	0	0	1	10.0	0	0	1	11.1	2	22.2	0	0	5	2.7	13	3.0
02_AG	0	0	4	6.6	0	0	0	0	0	0	0	0	0	0	1	11.1	0	0	0	0	11	6.0	16	3.7
F1	0	0	0	0	0	0	0	0	0	0	0	0	0	0	0	0	0	0	0	0	1	0.5	1	0.2
Other	0	0	6	9.8	3	14.3	0	0	0	0	1	10.0	0	0	2	22.2	0	0	1	12.5	19	10.3	32	7.4
Missing	74	75.5	12	19.7	9	42.9	5	45.5	4	40.0	5	50.0	6	60.0	4	44.4	7	77.8	6	75.0	110	59.8	242	56.1

LOQ: limit of quantification; MSM: men having sex with men; PWID: people who inject drugs; SD: standard deviation.

^a^ Biological sex at birth, as a binary variable. Information about transgenders was introduced in the InfCareHIV database in 2021 and is not presented in the table.

^b^ In patients with HIV RNA > LOQ only.

The demographics of Ukrainian PLWH differed from other major migrant groups in 2022 by being mostly female (60%) and infected heterosexually (56%) (Table 2). These differences also stood out in comparison with migrants from Ukraine before 2022, migrants from other FSU countries and from other countries that contributed most to migration to Sweden the past 5 years. To allow comparison of data that are beyond the scope of this manuscript, the Supplementary material provides a detailed, separate overview of the demographics of all (FSU) countries that contributed most HIV-1A6 cases (Supplementary Table S1), Ukrainian migrants in 2022 stratified by detectable HIV RNA (Supplementary Table S5) and migrants the past 5 years stratified by country (Supplementary Table S6).

The HIV-1 RNA load was determined in 95 of 98 Ukrainians migrating to Sweden in 2022; 76 of 98 (77%) self-reported to have received ART in Ukraine. Among the patients who reported being on ART, 10 had HIV-1 RNA above the limit of quantification (LOQ) at registration, most often after being without antiretrovirals for days or weeks.

**Figure 4:**
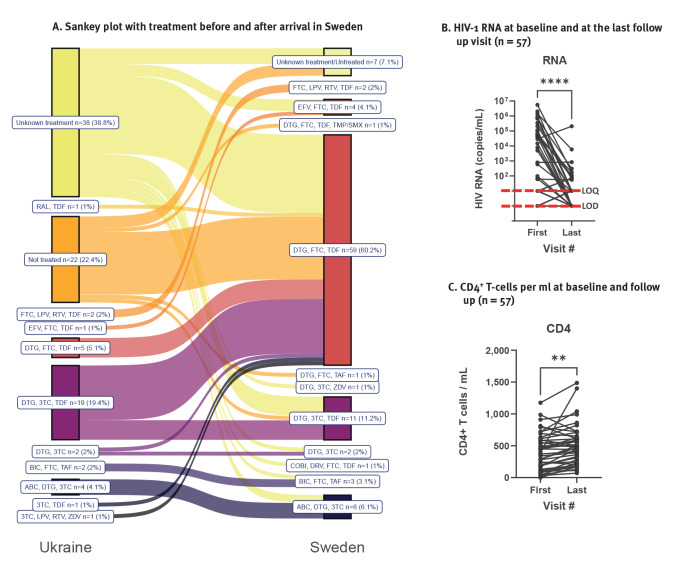
Treatment and outcome in people living with HIV who migrated from Ukraine to Sweden in 2022, Sweden, 1996–2022 (n = 98)

The most common treatment that was prescribed in Ukraine to 19 of the 38 PLWH with known treatment in our cohort, was a combination tablet with dolutegravir (DTG), lamivudine (3TC) and tenofovir disoproxil fumarate (TDF) (Figure 4A). Since this combination tablet DTG/3TC/TDF is not available in Sweden, most of these patients, as well as patients who could not report their ongoing treatment, were switched to the first-line therapy recommended in the Swedish National guidelines, which is DTG and emtricitabine (FTC)/TDF, unless there were contraindications. Three patients were on PI-based treatment, and one had an NNRTI-based regimen. Two patients from Ukraine had other treatment regimens that are not used in Sweden (3TC/TDF and RAL/TDF, respectively). Also these patients were switched to standard therapy after their first visit (Figure 4A).

3TC: lamivudine; ABC: abacavir; BIC: bictegravir; COBI: cobicistat; DRV: darunavir; DTG: dolutegravir; EFV: efavirenz; FTC: emtricitabine; LPV: lopinavir; RAL: raltegravir; RTV: ritonavir; TAF: tenofovir alafenamide; TDF: tenofovir disoproxil fumarate; TMP/SMX: trimethoprim/sulfamethoxazole; ZDV: zidovudine.

** p < 0.01.

**** p < 0.0001.

HIV-1 RNA was analysed at least twice in 57 patients, of whom 24 had an HIV RNA load above the LOQ, with an average follow-up duration of 130 days (standard deviation (SD): 79 days8). During follow up, the average RNA load decreased significantly (p < 0.0001) (Figure 4B). The number of PLWH with HIV-1 RNA above the LOQ of 50 copies/mL almost halved, from 24 to 13, and the number with undetectable HIV-1 RNA increased by 76%, from 17 to 30 (Figure 4B). There was a statistically significant increase in the average number of CD4^+^ T-cells from 517 cells/μL (SD: 344 cells/μL) to 577 cells/μL (SD: 351 cells/μL) between the first and last visit (p < 0.01) (Figure 4C). None of the patients experienced virological failure or switched drug regimen during the follow-up.

Among the seven PLWH in the ‘not treated/unknown treatment’ group, two had HIV RNA below the LOQ and follow-up in Sweden but no treatment was registered, two were viraemic without any treatment registered and emigrated before further follow-up in Sweden. Information on treatment was missing for three individuals.

Sequencing was possible in 24 of the 32 PLWH with HIV RNA above the LOQ and HIV-1A6 was identified in 23 of 24 sequences. This is a higher proportion than in Ukrainian PLWH arriving in Sweden before 2022 (25 of 36 where sequencing was possible).

## Discussion

The Swedish HIV-1 epidemic has evolved from being dominated by HIV-1B during the 1980 and 1990s to a diverse mixture of subtypes and recombinants [20]. Here, we show that this changing pattern has continued more recently, predominantly due to migration of PLWH to Sweden. Especially, the number of sequences that cannot be assigned to a (major) subtype has increased. As a large proportion of these sequences are recombinants, either CRFs or URFs, it is clear that for increasing numbers of patients, the different parts of the pol gene (protease, reverse transcriptase and integrase) may belong to different subtypes. This observation is of importance when considering the prescription of innovative chemotherapeutic (CAB/RPV) or antibody-based therapies, the effectiveness of which highly depends on the subtype of the integrase and the envelope gene, respectively. Thus, sequence analysis of at least the reverse transcriptase and integrase, but preferably also the envelope gene in all virologically presenting patients may not only be of use to assess drug resistance and monitor epidemiological trends but also as a cost-effective measure to inform prescribing innovative therapies.

HIV-1A6 is the most common subtype in the FSU and is consequently found in the majority of PLWH from this region. The number of new sequence-confirmed HIV-1A6 cases per year has increased in Sweden since the year 2000, and it is now the sixth most common subtype in Sweden. As only viraemic patients were sequenced, the real number of HIV-1A6 is certainly higher. We did not identify any clusters of HIV-1A6 transmission within Sweden, but several Swedish-born MSM infected with HIV-1A6 originated from an MSM cluster that was not related to a specific country of birth, country of infection or age group. HIV-1A6 infection is a risk factor for virological failure with long-acting injectable CAB/RPV therapy [10]. However, multidrug resistance was rare in PLWH with HIV-1A6 in Sweden, and only 14 of 208 sequences (6.7%) obtained between 2000 and 2022 had one or more major NRTI DRM, and 26 (12.5%) had at least one NNRTI DRM. This is lower than the percentage of HIV-1 isolates, regardless of subtype, carrying at least one DRM (18%) in untreated patients in Sweden during the period 2017 to 2019 [21].

Our 26-year surveillance of HIV-1 subtype distribution in Sweden illustrates the potential for a rapidly changing molecular HIV epidemiology within a geographical region, due to both migration and local transmission events.

In total 98 of the 431 (22.7%) new PLWH that entered HIV care in Sweden in 2022 were migrants from Ukraine. In comparison, only 67 PLWH migrated from Ukraine to Sweden between 1996 and December 2021. Most of the Ukrainian PLWH (66%) that came to Sweden in 2022 had HIV-1 RNA below the LOQ at enrolment in Swedish HIV care, which is comparable to previous studies from in Germany and Poland [22,23]. For 38 of 98 patients (39%), the previous treatment regime was identified by interview or review of previous prescriptions and/or medication. However, 38 (39%) lacked information of treatment history, probably because it was not registered by the treating physician. Twenty-two (22%) were registered as not previously treated. We found that half of the 38 patients (n = 19) were taking the combination tablet DTG/3TC/TDF, which is considerably less than the 75% of Ukrainian migrants reported to have previously received this therapy in Poland [23]. The discrepancy can be explained by the large proportion of Ukrainian PLWH with unknown treatment history in the Swedish cohort.

The majority of the Ukrainian PLWH who were enrolled in healthcare in Sweden in 2022 were aware of their HIV status and exhibited virological suppression and are therefore unlikely to contribute to further HIV spread within Sweden. However, 34% of them had detectable plasma HIV-1 RNA, and 17 individuals (18%) were diagnosed upon arrival in Sweden. Given the number of Ukrainian PLWH who were unaware of their status, and the voluntary nature of HIV testing on arrival, it seems likely that more Ukrainian migrants in Sweden may be infected with HIV-1 but not yet diagnosed. Late diagnosis poses a significant challenge in Swedish HIV care [24] as well as in Ukraine [25]. Indeed, we found that 27 (28%) of the Ukrainian PLWH arriving in 2022 presented with CD4^+^ T-cells < 350 cells/μL, which indicates late diagnosis also in many of the Ukrainian PLWH diagnosed before migrating to Sweden.

In general, the prevalence of HIV DRM in plasma virus was low in the Ukrainian PLWH registered in 2022 compared with newly diagnosed patients in the Swedish cohort as a whole. However, the presence of DRM in minor variants cannot be ruled out, especially in patients who experienced treatment interruption, as we did not perform resistance testing of peripheral blood mononuclear cells.

Several limitations in our data and analysis should be considered. The InfCareHIV database does not distinguish between refugees and other migrants , which may affect our findings with regard to the proportion of different subtypes among the Ukrainians diagnosed in Sweden. Ukrainian refugees in 2022 were more often heterosexually infected woman who are more frequently infected with HIV-1A6 than migrants in previous years who were more frequently MSM infected by HIV-1B. This may affect the percentage of people infected with subtypes or strains that are more prevalent in MSM. It should be noted that we are discussing all people who received care at a Swedish HIV care clinic between 1 January 1996 and 31 December 2022, and not the current population of PLWH in Sweden. Our estimate of the total number of HIV-1A6 patients who received care at some point in Sweden does not take in account demographic factors that may influence the odds that patients are tested and on treatment when arriving in Sweden, thus it should be considered a gross estimate. Records of HIV-1 RNA levels, CD4^+^ T-cell counts, DRM at diagnosis and previous treatment regimes may be incomplete in patients who were diagnosed or treated outside of Sweden. Treatment data are entered manually by the treating physician in smaller HIV care centres and may be incomplete in some cases. Our subtyping data are based on a limited part of the HIV genome, and thus we may underestimate the number of recombinants.

## Conclusion

Molecular HIV epidemiology in Sweden and other regions of the world is constantly evolving and heavily influenced by migration patterns, as illustrated by the recent increase in HIV-1A6 infections in Sweden related to migrants from Ukraine. Compared with other migrant groups in 2022, migrants from Ukraine were relatively more often heterosexually infected females. Although most Ukrainian PLWH arriving in Sweden had undetectable plasma viraemia, a substantial proportion of them were viraemic, had low CD4^+^ T-cell counts or were not aware of their HIV-1 infection. Consequently, there is a strong need to offer good HIV clinical care, and voluntary anonymous HIV testing should be offered and encouraged, both in the interest of the individual and society.

## Supplementary Data

658000 Supplement
