# Supplementary material for: The molecular epidemiology of HIV-1 in Sweden 1996 to 2022, and the influence of migration from Ukraine
Source: Euro Surveill. 2023 Nov 30;28(48):2300224. doi: 10.2807/1560-7917.ES.2023.28.48.2300224 (PMC10690863; doi:10.2807/1560-7917.ES.2023.28.48.2300224)
Supplement: Supplement [file 23-00224_vandeKLUNDERT_Supplement.pdf]

This supplementary material is hosted by Eurosurveillance as supporting information alongside the article “Molecular HIV-1 epidemiology in Sweden 1996-2022 and the influence of migration from Ukraine”, on behalf of the authors, who remain responsible for the accuracy and appropriateness of the content. The same standards for ethics, copyright, attributions and permissions as for the article apply. Supplements are not edited by Eurosurveillance and the journal is not responsible for the maintenance of any links or email addresses provided therein.

Supplementary Methods.

Geographical regions were assigned to the country of birth and country of infection using the United Nations standard country and area codes for statistical use. The sub regions “Sub-Saharan Africa” and “Latin America and the Caribbean” were further divided into their corresponding intermediate region (19). The countries of Taiwan (Eastern Asia), Kosovo (Southern Europe), and the former country “Former Yugoslavia” (Southern Europe) were added to their respective regions.

Figure S1

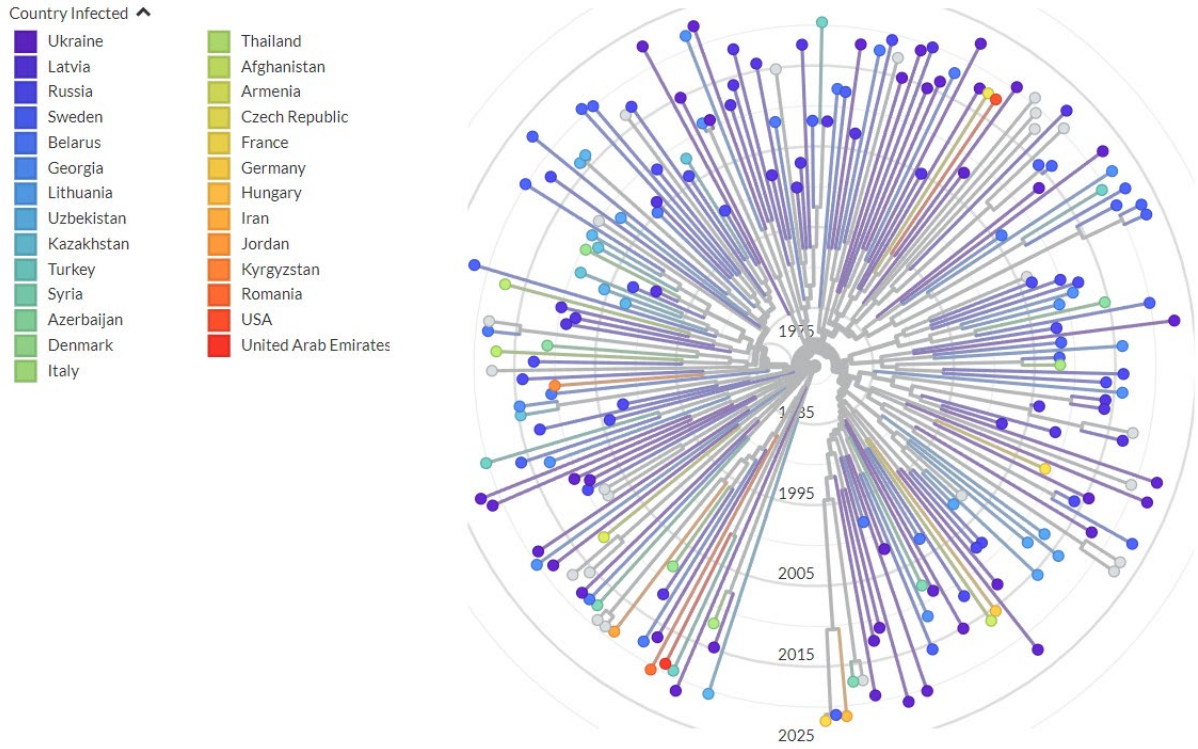

Phylogenetic tree of HIV-1A6 by country of infection (n=197)

TableS1

	Armenia (N=8)	Georgia (N=39)	Kazakhstan (N=12)	Latvia (N=50)	Lithuania (N=21)	Uzbekistan (N=32)	Russia (N=114)	Syria (N=54)	Ukraine (N=165)	Other (N=11159)	Overall (N=11654)
**Gender**											
Man	7 (87.5%)	33 (84.6%)	9 (75.0%)	38 (76.0%)	19 (90.5%)	23 (71.9%)	85 (74.6%)	48 (88.9%)	68 (41.2%)	7125 (63.8%)	7455 (64.0%)
Woman	1 (12.5%)	6 (15.4%)	3 (25.0%)	12 (24.0%)	2 (9.5%)	9 (28.1%)	29 (25.4%)	6 (11.1%)	95 (57.6%)	4021 (36.0%)	4184 (35.9%)
Missing	0 (0%)	0 (0%)	0 (0%)	0 (0%)	0 (0%)	0 (0%)	0 (0%)	0 (0%)	2 (1.2%)	13 (0.1%)	15 (0.1%)
**Age at diagnosis**											
Mean (SD)	32.4 (6.55)	36.6 (7.80)	35.2 (10.1)	31.6 (8.94)	27.3 (8.02)	32.6 (10.7)	31.3 (10.5)	34.2 (8.15)	34.2 (9.71)	35.4 (12.4)	35.3 (12.3)
Median [Min, Max]	30.0 [25.0, 41.0]	37.0 [23.0, 53.0]	32.0 [22.0, 54.0]	30.5 [16.0, 57.0]	28.0 [0, 42.0]	32.5 [8.00, 54.0]	31.0 [1.00, 57.0]	35.0 [17.0, 56.0]	33.0 [1.00, 63.0]	34.0 [-1.00, 107]	34.0 [-1.00, 107]
Missing	1 (12.5%)	1 (2.6%)	0 (0%)	2 (4.0%)	0 (0%)	2 (6.3%)	5 (4.4%)	2 (3.7%)	9 (5.5%)	258 (2.3%)	280 (2.4%)
**Infected in Sweden**											
Yes	1 (12.5%)	1 (2.6%)	1 (8.3%)	5 (10.0%)	3 (14.3%)	0 (0%)	20 (17.5%)	10 (18.5%)	6 (3.6%)	3025 (27.1%)	3072 (26.4%)
No	6 (75.0%)	37 (94.9%)	10 (83.3%)	44 (88.0%)	16 (76.2%)	29 (90.6%)	86 (75.4%)	37 (68.5%)	147 (89.1%)	7147 (64.0%)	7559 (64.9%)
Missing	1 (12.5%)	1 (2.6%)	1 (8.3%)	1 (2.0%)	2 (9.5%)	3 (9.4%)	8 (7.0%)	7 (13.0%)	12 (7.3%)	987 (8.8%)	1023 (8.8%)
**First RNA Load (x 1000 cp/mL)**											
Mean (SD)	128 (293)	96.1 (333)	132 (307)	261 (516)	267 (437)	671 (2210)	121 (367)	758 (2380)	141 (560)	864 (21900)	838 (21400)
Median [Min, Max]	13.0 [0, 791]	2.04 [0, 1900]	20.5 [0, 1080]	35.6 [0, 2160]	59.0 [0, 1700]	19.4 [0, 12200]	23.8 [0, 3500]	29.4 [0, 10000]	0.0400 [0, 5560]	27.2 [0, 1000000]	26.6 [0, 1000000]
Missing	1 (12.5%)	1 (2.6%)	0 (0%)	0 (0%)	1 (4.8%)	0 (0%)	4 (3.5%)	0 (0%)	4 (2.4%)	176 (1.6%)	187 (1.6%)
**First CD4 count**											
Mean (SD)	549 (321)	417 (256)	417 (235)	358 (229)	586 (990)	363 (203)	485 (331)	482 (276)	489 (307)	410 (316)	412 (318)
Median [Min, Max]	465 [200, 1170]	420 [10.0, 1130]	366 [130, 820]	340 [0, 1020]	352 [10.0, 4660]	374 [30.0, 860]	440 [14.0, 1990]	478 [50.0, 1510]	485 [0, 1730]	361 [0, 5730]	369 [0, 5730]
Missing	0 (0%)	1 (2.6%)	0 (0%)	1 (2.0%)	1 (4.8%)	0 (0%)	3 (2.6%)	0 (0%)	7 (4.2%)	139 (1.2%)	152 (1.3%)
**Subtype**											
A1	1 (12.5%)	0 (0%)	0 (0%)	0 (0%)	0 (0%)	0 (0%)	0 (0%)	2 (3.7%)	1 (0.6%)	549 (4.9%)	553 (4.7%)
A6	4 (50.0%)	14 (35.9%)	5 (41.7%)	26 (52.0%)	6 (28.6%)	6 (18.8%)	39 (34.2%)	5 (9.3%)	46 (27.9%)	58 (0.5%)	209 (1.8%)
B	1 (12.5%)	2 (5.1%)	2 (16.7%)	11 (22.0%)	3 (14.3%)	2 (6.3%)	21 (18.4%)	18 (33.3%)	5 (3.0%)	2454 (22.0%)	2519 (21.6%)
01_AE	0 (0%)	1 (2.6%)	0 (0%)	1 (2.0%)	1 (4.8%)	0 (0%)	1 (0.9%)	1 (1.9%)	1 (0.6%)	981 (8.8%)	987 (8.5%)
Other	0 (0%)	0 (0%)	4 (33.3%)	1 (2.0%)	1 (4.8%)	8 (25.0%)	5 (4.4%)	9 (16.7%)	1 (0.6%)	710 (6.4%)	739 (6.3%)
02_AG	0 (0%)	0 (0%)	0 (0%)	0 (0%)	0 (0%)	5 (15.6%)	1 (0.9%)	3 (5.6%)	0 (0%)	390 (3.5%)	399 (3.4%)
06_cpx	0 (0%)	0 (0%)	0 (0%)	0 (0%)	0 (0%)	0 (0%)	3 (2.6%)	2 (3.7%)	0 (0%)	43 (0.4%)	48 (0.4%)
F1	0 (0%)	0 (0%)	0 (0%)	0 (0%)	0 (0%)	0 (0%)	1 (0.9%)	0 (0%)	0 (0%)	25 (0.2%)	26 (0.2%)
G	0 (0%)	0 (0%)	0 (0%)	0 (0%)	0 (0%)	0 (0%)	1 (0.9%)	1 (1.9%)	0 (0%)	92 (0.8%)	94 (0.8%)
C	0 (0%)	0 (0%)	0 (0%)	0 (0%)	0 (0%)	0 (0%)	0 (0%)	1 (1.9%)	2 (1.2%)	1464 (13.1%)	1467 (12.6%)
D	0 (0%)	0 (0%)	0 (0%)	0 (0%)	0 (0%)	0 (0%)	0 (0%)	0 (0%)	2 (1.2%)	148 (1.3%)	150 (1.3%)
Missing	2 (25.0%)	22 (56.4%)	1 (8.3%)	11 (22.0%)	10 (47.6%)	11 (34.4%)	42 (36.8%)	12 (22.2%)	107 (64.8%)	4245 (38.0%)	4463 (38.3%)
**Transmission route**											
Blood products	0 (0%)	0 (0%)	0 (0%)	0 (0%)	0 (0%)	0 (0%)	3 (2.6%)	0 (0%)	5 (3.0%)	159 (1.4%)	167 (1.4%)
Heterosexual	2 (25.0%)	8 (20.5%)	4 (33.3%)	13 (26.0%)	3 (14.3%)	19 (59.4%)	37 (32.5%)	14 (25.9%)	76 (46.1%)	5445 (48.8%)	5621 (48.2%)
Mother-Child	0 (0%)	0 (0%)	0 (0%)	0 (0%)	1 (4.8%)	0 (0%)	3 (2.6%)	0 (0%)	2 (1.2%)	282 (2.5%)	288 (2.5%)
MSM	3 (37.5%)	9 (23.1%)	4 (33.3%)	15 (30.0%)	4 (19.0%)	8 (25.0%)	38 (33.3%)	35 (64.8%)	14 (8.5%)	3522 (31.6%)	3652 (31.3%)
PWID	1 (12.5%)	15 (38.5%)	1 (8.3%)	19 (38.0%)	9 (42.9%)	2 (6.3%)	18 (15.8%)	1 (1.9%)	17 (10.3%)	736 (6.6%)	819 (7.0%)
Unknown or Other	1 (12.5%)	3 (7.7%)	3 (25.0%)	2 (4.0%)	4 (19.0%)	2 (6.3%)	11 (9.6%)	4 (7.4%)	38 (23.0%)	753 (6.7%)	821 (7.0%)
Missing	1 (12.5%)	4 (10.3%)	0 (0%)	1 (2.0%)	0 (0%)	1 (3.1%)	4 (3.5%)	0 (0%)	13 (7.9%)	262 (2.3%)	286 (2.5%)

Table S1. Demographics of patients in the top-ten ranking birth countries of A6 infected PLWH in Sweden

TableS2

	<50 (N=6)	51-10000 (N=51)	10000-100000 (N=76)	>100000 (N=75)	Overall (N=208)
**First CD4 load**					
Mean (SD)	518 (264)	500 (237)	424 (261)	263 (283)	387 (280)
Median [Min, Max]	520 [90.0, 780]	460 [30.0, 990]	422 [20.0, 1510]	210 [0, 1990]	350 [0, 1990]
Missing	1 (16.7%)	0 (0%)	0 (0%)	0 (0%)	1 (0.5%)
**NRTI**					
None	6 (100%)	45 (88.2%)	67 (88.2%)	72 (96.0%)	190 (91.3%)
M184V	0 (0%)	1 (2.0%)	1 (1.3%)	0 (0%)	2 (1.0%)
S68G	0 (0%)	2 (3.9%)	1 (1.3%)	1 (1.3%)	4 (1.9%)
M184MV	0 (0%)	1 (2.0%)	1 (1.3%)	0 (0%)	2 (1.0%)
M184V,T215TFIS	0 (0%)	0 (0%)	0 (0%)	1 (1.3%)	1 (0.5%)
D67N,K70T,M184I	0 (0%)	0 (0%)	1 (1.3%)	0 (0%)	1 (0.5%)
V75VI	0 (0%)	0 (0%)	1 (1.3%)	0 (0%)	1 (0.5%)
S68N	0 (0%)	0 (0%)	1 (1.3%)	0 (0%)	1 (0.5%)
D67DN,L74LV,M184MV,K219KN	0 (0%)	0 (0%)	0 (0%)	1 (1.3%)	1 (0.5%)
L210W	0 (0%)	0 (0%)	1 (1.3%)	0 (0%)	1 (0.5%)
Missing	0 (0%)	2 (3.9%)	2 (2.6%)	0 (0%)	4 (1.9%)
**NNRTI**					
None	5 (83.3%)	44 (86.3%)	66 (86.8%)	63 (84.0%)	178 (85.6%)
G190S	0 (0%)	0 (0%)	1 (1.3%)	1 (1.3%)	2 (1.0%)
E138A	0 (0%)	2 (3.9%)	2 (2.6%)	3 (4.0%)	7 (3.4%)
K238N	0 (0%)	1 (2.0%)	0 (0%)	0 (0%)	1 (0.5%)
K103N	0 (0%)	1 (2.0%)	1 (1.3%)	1 (1.3%)	3 (1.4%)
A98G	1 (16.7%)	0 (0%)	0 (0%)	0 (0%)	1 (0.5%)
K101E,Y181C,G190S	0 (0%)	0 (0%)	1 (1.3%)	0 (0%)	1 (0.5%)
K103N,V106VI,H221HY,M230L,L234I	0 (0%)	0 (0%)	1 (1.3%)	0 (0%)	1 (0.5%)
V106VI	0 (0%)	0 (0%)	1 (1.3%)	2 (2.7%)	3 (1.4%)
V108VI	0 (0%)	1 (2.0%)	0 (0%)	0 (0%)	1 (0.5%)
Y181YC,G190GS,H221HY	0 (0%)	0 (0%)	0 (0%)	1 (1.3%)	1 (0.5%)
V179T	0 (0%)	0 (0%)	1 (1.3%)	0 (0%)	1 (0.5%)
G190GS	0 (0%)	0 (0%)	0 (0%)	1 (1.3%)	1 (0.5%)
V106VI,E138A	0 (0%)	0 (0%)	0 (0%)	1 (1.3%)	1 (0.5%)
E138AV	0 (0%)	0 (0%)	0 (0%)	1 (1.3%)	1 (0.5%)
K103KN	0 (0%)	0 (0%)	0 (0%)	1 (1.3%)	1 (0.5%)
Missing	0 (0%)	2 (3.9%)	2 (2.6%)	0 (0%)	4 (1.9%)
**PI Major**					
None	6 (100%)	49 (96.1%)	74 (97.4%)	75 (100%)	204 (98.1%)
M46I	0 (0%)	1 (2.0%)	2 (2.6%)	0 (0%)	3 (1.4%)
M46I,I54L,L90M	0 (0%)	1 (2.0%)	0 (0%)	0 (0%)	1 (0.5%)
**INSTI**					
None	5 (83.3%)	22 (43.1%)	24 (31.6%)	39 (52.0%)	90 (43.3%)
Missing	1 (16.7%)	29 (56.9%)	52 (68.4%)	36 (48.0%)	118 (56.7%)

Table S2. Overview of all drug resistance mutations (DRMs) identified in HIV-1A6 infected patients in Sweden stratified by HIV RNA cp/mL in plasma.

Table S3

NRTI	NNRTI	PI
**M184V**	**-**	**M46I, I54L, L90M**
**M184V T215TFIS**	**G190S**	**-**
**M184V**	**K103N, V106VI, H221HY, M230I**	**-**
**V74VI**	**E138A**	
	**V108VI**	**M46I, I54L, L90M**
**D76DN, L74LV, M184MV, K219KN**	**Y181YC, G190GS, H221HY**	**-**
**-**	**V106VI, E138A**	**-**
**L210W**	**V106VI**	**-**

Table S3. Combinations of multiple DRMs identified in people living with HIV-1A6

Table S4

	Overall (N=17)
**Gender**	
Man	6 (35.3%)
Woman	11 (64.7%)
**Age in years**	
Mean (SD)	41.1 (6.69)
Median [Min, Max]	42.0 [31.0, 56.0]
**First CD4+ T-cell count (cells/□l)**	
Mean (SD)	302 (296)
Median [Min, Max]	210 [0, 1180]
**First RNA Load (copies/ml)**	
Mean (SD)	454000 (1340000)
Median [Min, Max]	66000 [0, 5560000]
**Route of Transmission**	
Blood products	1 (5.9%)
Heterosexual	7 (41.2%)
Mother-Child	0 (0%)
MSM	0 (0%)
PWID	0 (0%)
Unknown or Other	9 (52.9%)

Table S4. Demographics newly diagnosed Ukrainians in Sweden 2022

HIV-1 RNA

HIV-1 RNA	Below LOQ		Above LOQ		Overall*	
**Total**						
Number (% Overall)	63	(66.3%)	32	(33.6%)	95	(100.0%)
**Gender**						
Male	22	(34.9%)	13	(40.6%)	35	(36.8%)
Female	39	(61.9%)	19	(59.4%)	58	(61.1%)
Missing	2	(3.2%)	0	(0%)	2	(2.1%)
**Age at diagnosis**						
Mean (SD)	33.9	(8.95)	37.5	(10.2)	35.2	(9.52)
Median [Min, Max]	32.5	[10.0, 51.0]	38.0	[12.0, 60.0]	35.0	[10.0, 60.0]
Missing	5	(7.9%)	1	(3.1%)	6	(6.3%)
**First CD4+ T-cell count (cells/µl)**						
Mean (SD)	626	(270)	361	(337)	533	(320)
Median [Min, Max]	600	[90.0, 1360]	265	[0, 1460]	510	[0, 1460]
Missing	4	(6.3%)	0	(0%)	4	(4.2%)
**Route of Transmission**						
Blood products	1	(1.6%)	3	(9.4%)	4	(4.2%)
Heterosexual	35	(55.6%)	15	(46.9%)	50	(52.6%)
Mother-Child	1	(1.6%)	0	(0%)	1	(1.1%)
MSM	3	(4.8%)	1	(3.1%)	4	(4.2%)
PWID	6	(9.5%)	1	(3.1%)	7	(7.4%)

HIV-1 RNA

HIV-1 RNA	Below LOQ		Above LOQ		Overall*	
Unknown or Other	12	(19.0%)	9	(28.1%)	21	(22.1%)
Missing	5	(7.9%)	3	(9.4%)	8	(8.4%)
**Treated at inclusion**						
Probably treated	24	(38.1%)	0	(0%)	24	(25.3%)
Treated	39	(61.9%)	10	(31.3%)	49	(51.6%)
Not treated	0	(0%)	22	(68.8%)	22	(23.2%)

Table S5. Demographics of Ukrainian immigrants in 2022 stratified by RNA load. LOQ: Limit of quantification (50 cp/ml)

Table S6

	Cameroon (N=42)	Eritrea (N=83)	Ethiopia (N=59)	Kongo (Kinshasa) (N=69)	Nigeria (N=53)	Sudan (N=32)	Thailand (N=113)	Uganda (N=53)	Ukraine (N=113)	Sweden (N=356)	Other (N=972)	Overall (N=1945)
**Gender***												
Man	25 (59.5%)	39 (47.0%)	23 (39.0%)	21 (30.4%)	22 (41.5%)	20 (62.5%)	24 (21.2%)	16 (30.2%)	41 (36.3%)	302 (84.8%)	702 (72.2%)	1235 (63.5%)
Woman	17 (40.5%)	44 (53.0%)	36 (61.0%)	47 (68.1%)	31 (58.5%)	12 (37.5%)	85 (75.2%)	37 (69.8%)	70 (61.9%)	54 (15.2%)	265 (27.3%)	698 (35.9%)
Missing	0 (0%)	0 (0%)	0 (0%)	1 (1.4%)	0 (0%)	0 (0%)	4 (3.5%)	0 (0%)	2 (1.8%)	0 (0%)	5 (0.5%)	12 (0.6%)
**Age at diagnosis**												
Mean (SD)	33.4 (10.7)	36.7 (14.8)	37.6 (13.1)	32.4 (13.0)	33.0 (11.2)	33.0 (10.2)	37.3 (11.2)	30.8 (15.2)	34.8 (9.57)	46.2 (15.2)	32.8 (12.3)	36.0 (13.8)
Median [Min, Max]	31.0 [18.0, 72.0]	38.0 [0, 68.0]	39.0 [3.00, 71.0]	34.0 [0, 55.0]	32.0 [2.00, 59.0]	30.0 [14.0, 65.0]	38.0 [6.00, 64.0]	30.0 [-1.00, 70.0]	34.0 [10.0, 60.0]	48.0 [0, 78.0]	31.0 [0, 77.0]	34.0 [-1.00, 78.0]
Missing	1 (2.4%)	6 (7.2%)	5 (8.5%)	2 (2.9%)	4 (7.5%)	1 (3.1%)	6 (5.3%)	3 (5.7%)	8 (7.1%)	9 (2.5%)	56 (5.8%)	101 (5.2%)
**Infected in Sweden or after arrival in Sweden**												
Yes	1 (2.4%)	7 (8.4%)	2 (3.4%)	0 (0%)	1 (1.9%)	1 (3.1%)	5 (4.4%)	1 (1.9%)	0 (0%)	152 (42.7%)	84 (8.6%)	254 (13.1%)
No	36 (85.7%)	66 (79.5%)	52 (88.1%)	63 (91.3%)	49 (92.5%)	31 (96.9%)	97 (85.8%)	51 (96.2%)	105 (92.9%)	151 (42.4%)	773 (79.5%)	1474 (75.8%)
Missing	5 (11.9%)	10 (12.0%)	5 (8.5%)	6 (8.7%)	3 (5.7%)	0 (0%)	11 (9.7%)	1 (1.9%)	8 (7.1%)	53 (14.9%)	115 (11.8%)	217 (11.2%)
**Transmission route**												
Blood products	0 (0%)	0 (0%)	0 (0%)	0 (0%)	1 (1.9%)	0 (0%)	1 (0.9%)	0 (0%)	4 (3.5%)	0 (0%)	10 (1.0%)	16 (0.8%)
Heterosexual	21 (50.0%)	56 (67.5%)	42 (71.2%)	47 (68.1%)	34 (64.2%)	13 (40.6%)	65 (57.5%)	34 (64.2%)	57 (50.4%)	160 (44.9%)	302 (31.1%)	831 (42.7%)
Mother-Child	0 (0%)	5 (6.0%)	3 (5.1%)	6 (8.7%)	2 (3.8%)	1 (3.1%)	1 (0.9%)	5 (9.4%)	1 (0.9%)	2 (0.6%)	32 (3.3%)	58 (3.0%)
MSM	13 (31.0%)	0 (0%)	1 (1.7%)	2 (2.9%)	7 (13.2%)	13 (40.6%)	21 (18.6%)	4 (7.5%)	5 (4.4%)	152 (42.7%)	449 (46.2%)	667 (34.3%)
PWID	0 (0%)	0 (0%)	0 (0%)	0 (0%)	0 (0%)	0 (0%)	0 (0%)	0 (0%)	9 (8.0%)	12 (3.4%)	36 (3.7%)	57 (2.9%)
Unknown or Other	0 (0%)	0 (0%)	0 (0%)	0 (0%)	0 (0%)	0 (0%)	0 (0%)	0 (0%)	0 (0%)	0 (0%)	0 (0%)	0 (0%)
Missing	8 (19.0%)	22 (26.5%)	13 (22.0%)	14 (20.3%)	9 (17.0%)	5 (15.6%)	25 (22.1%)	10 (18.9%)	37 (32.7%)	30 (8.4%)	143 (14.7%)	316 (16.2%)
**First CD4 T-cell count**												
Mean (SD)	421 (238)	343 (271)	425 (281)	462 (276)	454 (240)	375 (177)	293 (276)	462 (235)	517 (312)	387 (392)	485 (384)	444 (358)
Median [Min, Max]	393 [10.0, 920]	287 [10.0, 1310]	370 [10.0, 1180]	443 [10.0, 1200]	480 [10.0, 1000]	401 [30.0, 771]	232 [0, 1110]	460 [29.0, 1180]	500 [0, 1460]	360 [0, 5730]	450 [0, 4660]	410 [0, 5730]
Missing	0 (0%)	2 (2.4%)	0 (0%)	2 (2.9%)	0 (0%)	0 (0%)	1 (0.9%)	0 (0%)	6 (5.3%)	4 (1.1%)	27 (2.8%)	42 (2.2%)
**First RNA Load < 50 cp/mL**												
Yes	21 (50.0%)	27 (32.5%)	20 (33.9%)	38 (55.1%)	32 (60.4%)	12 (37.5%)	40 (35.4%)	31 (58.5%)	70 (61.9%)	35 (9.8%)	435 (44.8%)	761 (39.1%)
No	21 (50.0%)	55 (66.3%)	38 (64.4%)	30 (43.5%)	21 (39.6%)	19 (59.4%)	73 (64.6%)	22 (41.5%)	40 (35.4%)	320 (89.9%)	522 (53.7%)	1161 (59.7%)
Missing	0 (0%)	1 (1.2%)	1 (1.7%)	1 (1.4%)	0 (0%)	1 (3.1%)	0 (0%)	0 (0%)	3 (2.7%)	1 (0.3%)	15 (1.5%)	23 (1.2%)
**First RNA load (log10)****												
Mean (SD)	4.62 (1.38)	4.64 (1.22)	4.41 (1.25)	4.36 (1.17)	4.35 (0.971)	4.15 (1.13)	4.92 (1.08)	4.35 (1.47)	4.34 (1.38)	5.11 (1.22)	4.63 (1.27)	4.74 (1.27)
Median [Min, Max]	4.66 [2.08, 7.00]	4.60 [1.84, 8.00]	4.73 [1.76, 6.70]	4.45 [1.81, 6.62]	4.26 [2.47, 6.32]	4.48 [1.92, 5.60]	5.04 [1.80, 7.00]	4.78 [1.76, 6.08]	4.71 [1.74, 6.75]	5.14 [1.79, 9.00]	4.77 [1.72, 9.00]	4.85 [1.72, 9.00]
Missing	21 (50.0%)	28 (33.7%)	21 (35.6%)	39 (56.5%)	32 (60.4%)	13 (40.6%)	40 (35.4%)	31 (58.5%)	73 (64.6%)	36 (10.1%)	453 (46.6%)	787 (40.5%)
**Subtype**												
A6	0 (0%)	0 (0%)	1 (1.7%)	0 (0%)	0 (0%)	0 (0%)	0 (0%)	0 (0%)	29 (25.7%)	6 (1.7%)	52 (5.3%)	88 (4.5%)
C	1 (2.4%)	46 (55.4%)	33 (55.9%)	11 (15.9%)	2 (3.8%)	5 (15.6%)	2 (1.8%)	4 (7.5%)	1 (0.9%)	19 (5.3%)	70 (7.2%)	194 (10.0%)
B	0 (0%)	0 (0%)	0 (0%)	0 (0%)	0 (0%)	0 (0%)	7 (6.2%)	0 (0%)	1 (0.9%)	72 (20.2%)	92 (9.5%)	172 (8.8%)
02_AG	8 (19.0%)	1 (1.2%)	0 (0%)	1 (1.4%)	6 (11.3%)	1 (3.1%)	1 (0.9%)	1 (1.9%)	0 (0%)	22 (6.2%)	59 (6.1%)	100 (5.1%)
A1	2 (4.8%)	0 (0%)	0 (0%)	7 (10.1%)	1 (1.9%)	4 (12.5%)	1 (0.9%)	7 (13.2%)	0 (0%)	15 (4.2%)	46 (4.7%)	83 (4.3%)
G	1 (2.4%)	0 (0%)	1 (1.7%)	1 (1.4%)	10 (18.9%)	0 (0%)	0 (0%)	0 (0%)	0 (0%)	5 (1.4%)	8 (0.8%)	26 (1.3%)
01_AE	0 (0%)	2 (2.4%)	0 (0%)	1 (1.4%)	1 (1.9%)	0 (0%)	51 (45.1%)	0 (0%)	0 (0%)	102 (28.7%)	25 (2.6%)	182 (9.4%)
D	0 (0%)	4 (4.8%)	0 (0%)	0 (0%)	0 (0%)	3 (9.4%)	0 (0%)	5 (9.4%)	0 (0%)	1 (0.3%)	6 (0.6%)	19 (1.0%)
06_cpx	0 (0%)	0 (0%)	0 (0%)	0 (0%)	2 (3.8%)	1 (3.1%)	0 (0%)	0 (0%)	0 (0%)	0 (0%)	7 (0.7%)	10 (0.5%)
F1	0 (0%)	0 (0%)	0 (0%)	0 (0%)	0 (0%)	0 (0%)	0 (0%)	0 (0%)	0 (0%)	0 (0%)	4 (0.4%)	4 (0.2%)
Other	7 (16.7%)	0 (0%)	0 (0%)	8 (11.6%)	5 (9.4%)	5 (15.6%)	8 (7.1%)	2 (3.8%)	0 (0%)	52 (14.6%)	92 (9.5%)	179 (9.2%)
Missing	23 (54.8%)	30 (36.1%)	24 (40.7%)	40 (58.0%)	26 (49.1%)	13 (40.6%)	43 (38.1%)	34 (64.2%)	82 (72.6%)	62 (17.4%)	511 (52.6%)	888 (45.7%)

Table S6, demographics and virological characteristics of PLWH migrating to Sweden 2018 - 2022 by country

Fig. S7 – Copy of Table 2

	Ukraine (N=98)	Sweden (N=61)	Thailand (N=21)	Ethiopia (N=11)	Eritrea (N=10)	Kongo (Kinshasa) (N=10)	Russia (N=10)	Cameroon (N=8)	Sudan (N=9)	Uganda (N=9)	Other (N=184)	Overall (N=431)
**Sex***												
Man	36 (36.7%)	49 (80.3%)	5 (23.8%)	6 (54.5%)	4 (40.0%)	4 (40.0%)	9 (90.0%)	7 (87.5%)	6 (66.7%)	1 (11.1%)	124 (67.4%)	251 (58.2%)
Woman	60 (61.2%)	12 (19.7%)	12 (57.1%)	5 (45.5%)	6 (60.0%)	6 (60.0%)	1 (10.0%)	1 (12.5%)	3 (33.3%)	8 (88.9%)	55 (29.9%)	169 (39.2%)
Missing	2 (2.0%)	0 (0%)	4 (19.0%)	0 (0%)	0 (0%)	0 (0%)	0 (0%)	0 (0%)	0 (0%)	0 (0%)	5 (2.7%)	11 (2.6%)
**Age at diagnosis**												
Mean (SD)	35.4 (9.72)	45.8 (14.4)	38.2 (14.6)	39.8 (12.2)	38.7 (14.5)	33.6 (15.6)	28.3 (9.98)	36.5 (9.43)	30.6 (9.79)	32.0 (16.1)	33.5 (12.7)	36.1 (13.1)
Median [Min, Max]	35.0 [10.0, 60.0]	47.5 [20.0, 69.0]	34.0 [17.0, 64.0]	40.0 [8.00, 52.0]	41.0 [5.00, 56.0]	37.0 [0, 51.0]	26.5 [17.0, 42.0]	32.5 [27.0, 52.0]	29.0 [14.0, 47.0]	33.0 [4.00, 58.0]	32.0 [0, 77.0]	34.0 [0, 77.0]
Missing	7 (7.1%)	5 (8.2%)	2 (9.5%)	1 (9.1%)	0 (0%)	1 (10.0%)	2 (20.0%)	0 (0%)	0 (0%)	2 (22.2%)	21 (11.4%)	41 (9.5%)
**Infected in Sweden or after arrival in Sweden**												
Yes	0 (0%)	34 (55.7%)	2 (9.5%)	0 (0%)	1 (10.0%)	0 (0%)	2 (20.0%)	0 (0%)	1 (11.1%)	0 (0%)	18 (9.8%)	58 (13.5%)
No	91 (92.9%)	20 (32.8%)	18 (85.7%)	11 (100%)	7 (70.0%)	9 (90.0%)	6 (60.0%)	7 (87.5%)	8 (88.9%)	9 (100%)	146 (79.3%)	332 (77.0%)
Missing	7 (7.1%)	7 (11.5%)	1 (4.8%)	0 (0%)	2 (20.0%)	1 (10.0%)	2 (20.0%)	1 (12.5%)	0 (0%)	0 (0%)	20 (10.9%)	41 (9.5%)
**Transmission route**												
Blood products	4 (4.1%)	0 (0%)	0 (0%)	0 (0%)	0 (0%)	0 (0%)	0 (0%)	0 (0%)	0 (0%)	0 (0%)	4 (2.2%)	8 (1.9%)
Heterosexual	50 (51.0%)	32 (52.5%)	11 (52.4%)	8 (72.7%)	7 (70.0%)	5 (50.0%)	2 (20.0%)	3 (37.5%)	2 (22.2%)	5 (55.6%)	60 (32.6%)	185 (42.9%)
Mother-Child	1 (1.0%)	0 (0%)	0 (0%)	1 (9.1%)	1 (10.0%)	2 (20.0%)	0 (0%)	0 (0%)	1 (11.1%)	1 (11.1%)	4 (2.2%)	11 (2.6%)
MSM	4 (4.1%)	23 (37.7%)	5 (23.8%)	0 (0%)	0 (0%)	1 (10.0%)	3 (30.0%)	5 (62.5%)	5 (55.6%)	0 (0%)	76 (41.3%)	122 (28.3%)
PWID	7 (7.1%)	0 (0%)	0 (0%)	0 (0%)	0 (0%)	0 (0%)	2 (20.0%)	0 (0%)	0 (0%)	0 (0%)	0 (0%)	9 (2.1%)
Unknown or Other	0 (0%)	0 (0%)	0 (0%)	0 (0%)	0 (0%)	0 (0%)	0 (0%)	0 (0%)	0 (0%)	0 (0%)	0 (0%)	0 (0%)
Missing	32 (32.7%)	6 (9.8%)	5 (23.8%)	2 (18.2%)	2 (20.0%)	2 (20.0%)	3 (30.0%)	0 (0%)	1 (11.1%)	3 (33.3%)	40 (21.7%)	96 (22.3%)
**First CD4 T-cell count**												
Mean (SD)	530 (320)	371 (271)	289 (278)	457 (251)	342 (261)	377 (358)	601 (295)	530 (148)	472 (224)	493 (193)	480 (272)	464 (287)
Median [Min, Max]	505 [0, 1460]	340 [2.00, 1000]	218 [0, 1110]	430 [170, 990]	230 [100, 850]	240 [120, 1120]	800 [39.0, 920]	534 [320, 780]	450 [67.0, 771]	510 [164, 704]	470 [0, 1310]	450 [0, 1460]
Missing	6 (6.1%)	3 (4.9%)	0 (0%)	0 (0%)	1 (10.0%)	2 (20.0%)	1 (10.0%)	0 (0%)	0 (0%)	0 (0%)	15 (8.2%)	28 (6.5%)
**First RNA Load < 50 cp/mL**												
Yes	63 (64.3%)	6 (9.8%)	9 (42.9%)	4 (36.4%)	3 (30.0%)	4 (40.0%)	3 (30.0%)	6 (75.0%)	4 (44.4%)	7 (77.8%)	93 (50.5%)	202 (46.9%)
No	32 (32.7%)	54 (88.5%)	12 (57.1%)	7 (63.6%)	6 (60.0%)	5 (50.0%)	6 (60.0%)	2 (25.0%)	5 (55.6%)	2 (22.2%)	84 (45.7%)	215 (49.9%)
Missing	3 (3.1%)	1 (1.6%)	0 (0%)	0 (0%)	1 (10.0%)	1 (10.0%)	1 (10.0%)	0 (0%)	0 (0%)	0 (0%)	7 (3.8%)	14 (3.2%)
**First RNA load (log10)****												
Mean (SD)	4.29 (1.49)	5.09 (1.13)	4.62 (1.41)	4.33 (1.23)	4.90 (1.83)	4.39 (0.532)	3.53 (1.34)	3.94 (2.63)	3.67 (1.51)	4.79 (1.56)	4.60 (1.41)	4.62 (1.38)
Median [Min, Max]	4.71 [1.74, 6.75]	5.38 [1.88, 6.98]	4.99 [1.90, 6.19]	4.79 [1.76, 5.49]	4.42 [2.80, 8.00]	4.43 [3.77, 4.94]	3.95 [1.72, 5.00]	3.94 [2.08, 5.80]	4.09 [1.93, 5.60]	4.79 [3.69, 5.90]	4.82 [1.75, 7.00]	4.83 [1.72, 8.00]
Missing	66 (67.3%)	7 (11.5%)	9 (42.9%)	4 (36.4%)	4 (40.0%)	5 (50.0%)	4 (40.0%)	6 (75.0%)	4 (44.4%)	7 (77.8%)	101 (54.9%)	217 (50.3%)
**Subtype**												
A6	23 (23.5%)	3 (4.9%)	0 (0%)	0 (0%)	0 (0%)	0 (0%)	3 (30.0%)	0 (0%)	0 (0%)	0 (0%)	3 (1.6%)	32 (7.4%)
B	1 (1.0%)	14 (23.0%)	1 (4.8%)	0 (0%)	0 (0%)	0 (0%)	1 (10.0%)	0 (0%)	0 (0%)	0 (0%)	16 (8.7%)	33 (7.7%)
G	0 (0%)	1 (1.6%)	0 (0%)	0 (0%)	0 (0%)	1 (10.0%)	0 (0%)	1 (12.5%)	0 (0%)	0 (0%)	0 (0%)	3 (0.7%)
C	0 (0%)	2 (3.3%)	1 (4.8%)	6 (54.5%)	4 (40.0%)	2 (20.0%)	0 (0%)	0 (0%)	0 (0%)	0 (0%)	13 (7.1%)	28 (6.5%)
D	0 (0%)	0 (0%)	0 (0%)	0 (0%)	1 (10.0%)	0 (0%)	0 (0%)	0 (0%)	1 (11.1%)	0 (0%)	1 (0.5%)	3 (0.7%)
01_AE	0 (0%)	15 (24.6%)	7 (33.3%)	0 (0%)	1 (10.0%)	0 (0%)	0 (0%)	0 (0%)	0 (0%)	0 (0%)	5 (2.7%)	28 (6.5%)
A1	0 (0%)	4 (6.6%)	0 (0%)	0 (0%)	0 (0%)	1 (10.0%)	0 (0%)	0 (0%)	1 (11.1%)	2 (22.2%)	5 (2.7%)	13 (3.0%)
02_AG	0 (0%)	4 (6.6%)	0 (0%)	0 (0%)	0 (0%)	0 (0%)	0 (0%)	0 (0%)	1 (11.1%)	0 (0%)	11 (6.0%)	16 (3.7%)
F1	0 (0%)	0 (0%)	0 (0%)	0 (0%)	0 (0%)	0 (0%)	0 (0%)	0 (0%)	0 (0%)	0 (0%)	1 (0.5%)	1 (0.2%)
Other	0 (0%)	6 (9.8%)	3 (14.3%)	0 (0%)	0 (0%)	1 (10.0%)	0 (0%)	1 (12.5%)	2 (22.2%)	0 (0%)	19 (10.3%)	32 (7.4%)
Missing	74 (75.5%)	12 (19.7%)	9 (42.9%)	5 (45.5%)	4 (40.0%)	5 (50.0%)	6 (60.0%)	6 (75.0%)	4 (44.4%)	7 (77.8%)	110 (59.8%)	242 (56.1%)

Table S7, Copy of Table 2 with more convenient formatting for evaluation. demographics and virological characteristics of PLWH migrating to Sweden in 2022 by country
